# Use of Waste Red Seaweed Furcellaran for Development of Green Thermoplastically Processable Bioplastics

**DOI:** 10.3390/polym18070884

**Published:** 2026-04-04

**Authors:** Remo Merijs-Meri, Jānis Zicāns, Tatjana Ivanova, Juris Bitenieks, Pēteris Patriks Jefimovs, Ivans Bočkovs, Žanis Edvards Rībens, Rita Bērziņa, Aina Bernava, Reina Rozentāle, Karina Bāliņa, Uldis Žaimis

**Affiliations:** 1 Institute of Chemistry and Chemical Technology, Faculty of Natural Sciences and Technology, Riga Technical University, 3 Paula Valdena Street, LV-1048 Riga, Latvia; 2 Centre of Science and Engineering, 14 Lielā Street, RTU Liepaja, LV-3401 Liepaja, Latvia

**Keywords:** furcellaran, thermoplastic starch, thermal properties, rheological properties, mechanical properties

## Abstract

Bioplastics are in focus for the development of sustainable materials due to the depletion of fossil resources, generation of solid waste and global climate change. Considering this, the current research is devoted to the valorization of beachcast red seaweed *F. lumbricalis* for the development of thermoplastically processable bioplastics. The composites have been developed from beachcast red seaweed-derived furcellaran (FUR) and potato-derived thermoplastic starch (TPS) by using an ultrasound-assisted technique. Three different FUR concentrations (10, 30 and 50 wt.%) in relation to potato starch were examined for their thermoplastic processability. Fourier infrared spectroscopy (FTIR) was used to reveal the structural changes in the developed TPS/FUR composites depending on FUR content as well as thermal pre-treatment. Thermogravimetric analysis (TGA), differential scanning calorimetry (DSC), and tensile mechanical tests were performed to assess the performance of the developed TPS/FUR composites. It was demonstrated that the ultrasound-assisted manufacturing route allowed TPS/FUR composites with an improved spectrum of properties to be obtained. The highest mechanical stress at break (almost three times higher than for neat TPS) was observed for the TPS + 50 wt.% FUR composite, which also possessed decreased deformability (only ca 10%), reduced thermal resistance at processing temperatures (150 °C) and high shear sensitivity. Thus, the TPS + 30 wt.% FUR and especially the TPS + 10 wt.% FUR composites were recognized as more suitable for thermoplastic processing and the development of TPS-based composites with improved exploitation properties.

## 1. Introduction

Algae are a polyphyletic assembly of main photosynthetic life forms, usually found in water bodies, which vary between microscopic single-celled types to multicellular seaweeds [1]. These organisms have critical functions in terrestrial and marine biogeochemical processes; they are estimated to generate over half of the photosynthetic capacity of the world, and they form the foundation of aquatic food webs [2]. Algae do not have true roots, stems, and leaves as in higher plants (thallus character) but assume a simple filamentous form or a complex parenchymatous one. There is more to algae’s contribution to ecology than just primary production [3], as they are also involved in nitrogen fixation, play a major role in sediment formation and provide important habitat structures in the sea. Using algae in the production of biofuels, nutraceuticals and carbon sequestration technology is a recent development in biotechnology considering the rapid growth rates of algae, which are capable of doubling themselves within 24 h under optimum conditions [4].

Marine macroalgae species may be classified within three main groups, including the green algae *Chlorophyta*, red algae *Rhodophyta* and brown algae *Phaeophyceae*. Of the red algal species, *F. lumbricalis*, abundant in the Baltic Sea area, is a vital source of furcellaran (FUR), a sulphated polysaccharide with a chemical structure similar to carrageenan. In addition to furcellaran, which ensures good gelling properties, *F. lumbricalis* contains 10–15% cellulose, 5–10% proteins, and 15–25% minerals (primarily potassium K^+^ and calcium Ca^2+^ ions) [5], and is thus a potential candidate for the development of new multifunctional, biobased materials, including bioplastics. Although *F. lumbricalis* has already been used commercially, mainly for the extraction of FUR to produce food and beauty industry additives, part of this red seaweed species is wasted as FUR industry byproducts or naturally occurring beachcast algal waste, annually generated due to seawater exchange, especially after storm events. As an example, the volume of beachcast seaweed on Liepāja beach (Latvia) typically exceeds 200 m^3^ per 100 m, especially during the autumn season [6]. Unfortunately, beachcast algal waste is presently seldom utilized in a sustainable way, mainly being disposed of in landfill.

Consequently, there is an interest in the valorization of beachcast algal waste, as in the current best-case scenario it is composted [7]. Besides this, disregarding the large potential usage of macroalgae in biomedicine, the health and beauty sector, the food and packaging industry, energetics and environmental engineering [8,9,10,11,12,13,14,15], there are practically no scientific investigations on the development of macroalgae-based melt-processable bioplastics, in spite of the wide industrial acceptance of thermoplastic processing for production of a broad spectrum of consumer products. This led to the idea of using the beachcast red seaweed *F. lumbricalis* for developing new furcellaran-containing bioplastic composites.

There are several approaches which can be used to improve the thermoplastic processability of polymers, such as adding a plasticizer, stabilizing a melt with processing aids and blending with other melt-processable polymers. For the development of green thermoplastic polymer composites, priority is often given to industrially produced biobased and biodegradable high-molecular components with known thermoplastic processing behavior such as polylactide (PLA), polyhydroxyalkanoate (PHA), polybutylene succinate (PBS) and thermoplastic starch (TPS) blends [6]. Although all the mentioned polymers are biodegradable, the rate of their biodegradation differs depending on the external environment (e.g., soil, compost, fresh water, saline water). In general, it has been agreed that the biodegradation rate of these polymers increases as follows: 13% over 60 days for PLA, 90% over 160 days for PBS, 85% over 90 days for starch-based blends and 80% over 28 days for polyhydroxybutyrate under the industrial compost conditions [16]. Concomitantly, the biodegradation of starch-based films may be increased even more by the addition of carrageenan, which has a similar molecular structure to FUR; it is believed that faster biodegradation occurs due to α-1,3 and β-1,4-glycosidic linkages between monomeric units of carrageenan [17,18]. From the previously listed commercially most important biobased and biodegradable polymers, starch-based bioplastics stand out due to their availability and large amounts of low-cost resources, which compete less with the resources necessary for the food industry as in the cases of PLA and PBS, lower production costs in comparison to PHA, and total biodegradability in comparison to PBS, for which biobased content usually is smaller than for its counterparts. In addition, it is interesting to mention that the price of bioplastics may be reduced and their competitiveness with fossil-based plastics may also be increased by using biomass residues; for example, beachcast algae or industrial algal waste for extraction of FUR or fruit peels, and plant seeds, leaves or even food processing industry wastewater [19,20,21] for obtaining TPS.

Consequently, TPS, obtained from potatoes, was chosen as the thermoplastic matrix for the development of composites with FUR, which was extracted from beachcast red seaweed. The aim was to assess the amount of FUR that can be added to TPS while maintaining its processability and ensuring improved mechanical and thermal properties. We have assumed that the addition of certain amounts of FUR to TPS will help to improve the stiffness and strength of the developed composites without considerably decreasing the melt-processability of TPS.

## 2. Materials and Methods

### 2.1. Raw Materials

Potato starch from Aloja-Starkelsen SIA (Ungurpils, Latvia) was used. According to Almonaityte [22], the starch contained 21–23 wt.% of amylose and 77–79 wt.% of amylopectin, and had an intrinsic viscosity of 0.39 L/g and a molecular weight of 103–104 kDa. Commercial glycerol and distilled water were used as plasticizers and rheology modifiers.

Furcellaran (FUR) was obtained from Est-Agar AS (Kärla, Estonia). According to the manufacturer, the commercial carrageenan consisted of beige to brownish yellow powder with greyish shade, a neutral taste and odor. It was expected to be soluble in hot water and insoluble in alcohol for a 1.5% dilution. Loss on drying: ≤12%. Particle size: at least 95% <150 μm.

### 2.2. Preparation of Composites and Test Specimens

To obtain neat plasticized starch, the dried raw potato starch was manually mixed with glycerol and distilled water with a specific ratio by weight (5:3.3:2). In the case of FUR-containing systems (10, 30 and 50 wt.% in respect to the starch replacement amount) it was, however, necessary, to increase the water amount, making the ratio between starch, glycerol and water 5:3.3:5. This was due to increased viscosity caused by the strong gelling properties of FUR. After this initial pre-mixing step, the vessel with the prepared pre-mixture was placed in a Sonorex Digiplus ultrasonic bath (Bandelin electronic GmbH & Co. KG, Berlin, Germany) and was further processed by using ultrasound at 35 kHz until the beginning of gelation at ca 80 °C. After sonification, the dough-like mass of the plasticized starch or its blend with FUR was processed by using two-roll mills LRM-S-110/3E (Labtech Engineering Co., Ltd., Phraeksa, Thailand) at a friction of 1.25 and roll temperatures of 140 and 135 °C. The prepared TPS/FUR composites were carefully placed into the frame mold of a LP-S-50/S.ASTM hydraulic press (Labtech Engineering Co., Ltd., Phraeksa, Thailand) to obtain plates with lateral dimensions of 10 cm × 10 cm. The thickness of the plates was 0.5 mm for obtaining tensile test specimens and 1 mm for obtaining test specimens for rheology tests. The compression molding was carried out at a temperature of 140 °C for a duration of 3 min. The samples were stored in a desiccator with freshly pre-heated calcium chloride CaCl_2_ to avoid absorbing moisture from the environment until the tests were performed.

### 2.3. Functional Group Analysis

Functional group analysis was carried out by taking Fourier infrared spectroscopy spectra. The spectra were taken using a Nicolet 6700 spectrometer (Thermo Fisher Scientific, Waltham, MA, USA) via the attenuated total reflectance (ATR) accessory. All spectra were taken in the range of 650 to 4000 cm^−1^ with a resolution of 4 cm^−1^.

### 2.4. Testing of Tensile Properties

Tensile properties of neat TPS and its composites with FUR were tested using a Universal Testing Machine ST25 (Tinius Olsen, Horsham, PA, USA) equipped with a 5 kN Z-type load cell. Initial grip-to-grip distance was 40 mm, whereas gage length was 30 mm. Testing speed was 50 mm/min. Testing was performed at 25 °C and 50% RH. Five dumbbell test specimens, cut from the compression-molded plates, were tested. Deviation of the individual measurement from average results was below 10 and 20% for stress and strain measurements, respectively.

### 2.5. Testing of Rheological Properties

The rheological properties of neat TPS and its composites with FUR were examined using a SmartPave 102 DSR rheometer (Anton Paar GmbH, Graz, Austria) in plate–plate configuration. The plate diameter was 25 mm and the gap size was 1 mm. Testing temperature was 150 °C. Disc-shaped test specimens were cut from the compression-molded plates. Initially, the measurements were performed by changing shear strain amplitude at an oscillation rate equal to 1 Hz to determine the linear viscoelasticity region (LVER). At the determined shear strain, frequency scan tests were performed. After the frequency scan test, the LVER test was repeated to evaluate complex viscosity change due to the thermal ageing of the test specimen. In addition, flow curves were measured over the shear rate range 0.01–100 s^−1^. All the measurements were made in duplicate.

### 2.6. Testing of Calorimetric Properties

Differential scanning calorimetry (DSC) of neat TPS and its composites with FUR was performed using the DSC-3+ apparatus (Mettler Toledo, Greifensee, Switzerland). The typical sample size for the measurements was ca 10 mg. The DSC test was performed in an inert atmosphere under a N_2_ gas flow rate of 50 mL/min. To remove thermal pre-history, test specimens were heated from −90 to 200 °C at a heating rate of 10 °C/min (marked with abbreviation r1), followed by holding at 200 °C for 5 min, cooling back to −90 °C at the same rate of 10 °C/min (marked with abbreviation c) and holding at −90 °C for 5 min, then finished by a second heating up to 200 °C, maintaining the same rate (marked with abbreviation r2).

### 2.7. Testing of Thermogravimetric Properties

Thermogravimetric analysis (TGA) of neat TPS and its composites with FUR was performed using the TGA/DSC3+ apparatus (Mettler Toledo, Greifensee, Switzerland). The typical sample size for the measurements was ca 10 mg. The TGA test was performed in an oxidative atmosphere under an air flow rate of 50 mL/min. The heating was performed from 25 up to 800 °C at a heating rate of 10 °C.

## 3. Results

### 3.1. Fourier Infrared Spectroscopy

**Figure 1:**
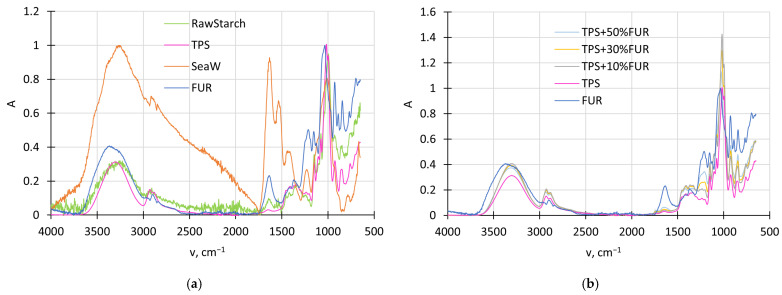
FTIRATR spectra of the red seaweed, FUR, raw potato starch, TPS (**a**), and its composites (**b**).

By considering the effect that thermal processing can have on the structures of natural materials, the FTIR-ATR spectra of neat potato starch and cold-water red seaweed *F. lumbricalis* were compared with their thermally processed descendants, TPS and FUR, respectively. As demonstrated in Figure 1a, the spectrum of the red seaweeds is characterized by a broad multimodal peak resulting from overlapping of the individual peaks of the components present in the seaweed. It has been previously determined that *F. lumbricalis* is rich in proteins (28%) and contains relatively high amounts of phenolic compounds (3.25%) and monounsaturated fatty acids (29%). Concomitantly, FUR content in red seaweed can fluctuate within the 19–50% range [23].

Consequently, after the extraction, the characteristic peak intensity decreased over the broad diapason of 1750–4000 cm^−1^, revealing characteristic peaks of FUR centered around 2900 and 3335 cm^−1^, which are attributed to C-H bond stretching and hydroxyl group vibrations, respectively. In the wavenumber region between 1300 and 1750 cm^−1^, the broad multimodal peak of the red seaweed has transformed into two expressed peaks centered around 1650 and 1360 cm^−1^, which are assigned to the bending oscillations of –OH groups of the absorbed water molecules and ester sulphate absorption bands, respectively. The FUR extract also demonstrates characteristic ester sulphate vibrations centered at 1210 cm^−1^. This testifies that the FUR component has been successfully extracted from the red seaweed. In turn, the intensive peaks around 950–1075 cm^−1^ are assigned to the skeleton of galactans and the vibrations of the C–O–C bands, whereas the peak centered at 850 cm^−1^ is assigned to the galactose 4-sulphate units of FUR [24]. From the spectra, it is evident that the chemical structure of FUR is not considerably affected during its extraction from the red seaweeds.

Figure 1a also reveals the differences between the raw potato starch and TPS. The most prominent differences are observed at 2854 cm^−1^, corresponding to the stretching of the C–H bond, as well as in the region of 1500–1750 cm^−1^, where the intensity of the characteristic peaks of native potato starch has reduced, forming a single peak centered at 1650 cm^−1^, which can be attributed to the addition of the glycerol plasticizer and the presence of the starch-bound water molecules [25]. The shift in the absorption bands to the higher wavenumber values within the range of 950 and 1050 cm^−1^ and the increased peak intensity are associated with the gelatinization of the native starch.

After development of TPS/FUR composites, the largest changes are observed in the wavenumber regions from 1550 to 1760 cm^−1^, centered around 1650 cm^−1^, and from 1180 to 1290 cm^−1^, centered around 1210 cm^−1^, which are assigned to the –OH groups of the absorbed water molecules due to the presence of TPS and FUR, and ester sulphate absorption due to the presence of FUR (Figure 1b). Moreover, by increasing FUR content in the TPS matrix, the absorption bands within 900–1300 cm^−1^, 1550–1760 cm^−1^ and 3000–3750 cm^−1^ have been transformed, evidently because of the development of hydrogen H bonds between the C−O−H and C−O−C groups, found in the starch structure and hydroxyl moieties in the furcellaran.

Simultaneously, attention should be paid to the wavenumber range between 3550 and 3200 cm^−1^, which represents the intermolecular bonding of the hydroxyl groups, as well as the wavenumber range between 3200 and 2700 cm^−1^, which, in turn, represents the intramolecular bonding [26] of the hydroxyl groups, revealing that for the investigated composites, intramolecular interaction becomes more important if FUR content is increased.

The slight increment in intensity around 1740 cm^−1^ may denote the beginning of thermo-oxidation processes, especially at the highest FUR content; moreover, the relatively larger decomposition of TPS + 50 wt.% FUR composites is confirmed by the peak emerging around 890 cm^−1^, which was not observed for other TPS/FUR composites. This results in a decreased ratio between the peaks around 930 and 890 cm^−1^. The decreased ratio of these signals (A930/A890) was found to be characteristic of more degraded samples due to the lower stability of the 3,6-anhydrogalactopyranose residues in FUR in comparison to the β-galactose residues [27].

### 3.2. Thermogravimetric Analysis

**Figure 2:**
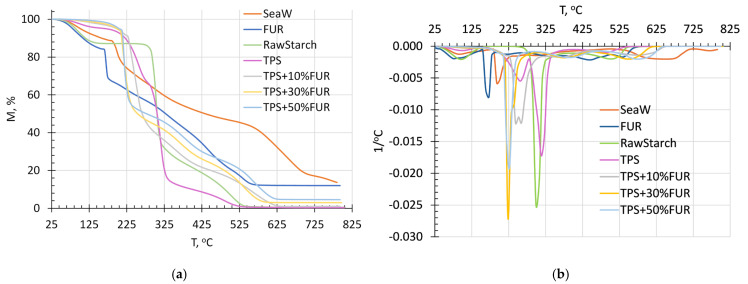
Thermogravimetric analysis curves of the raw starch, waste red seaweed, FUR, TPS and TPS/FUR composites: mass loss–temperature relationships (**a**) and 1st derivative against temperature–temperature relationships (**b**).

The thermal stability of the raw potato starch, waste red seaweed, TPS, FUR, and the investigated TPS/FUR composites is characterized by the TGA thermograms shown in Figure 2.

It can be clearly seen that the raw potato starch, the waste red seaweed and particularly the FUR extracted from the red seaweed are sensitive to temperature. A remarkable exothermic effect of FUR is observed close to 150 °C, which therefore should be considered as the maximum allowable thermoplastic processing temperature of TPS/FUR composites. The higher thermal stability of the seaweed in comparison to FUR is due to the presence of more thermally resistant components, e.g., cellulose, proteins, and minerals [5].

By examining the effect of FUR on the thermal stability of TPS, a complicated multistep thermodegradation pattern can be observed (Figure 2a). However, it can also be seen that some of the thermo-oxidative degradation steps overlap. For a more detailed analysis, the first derivative curves against temperature are shown in Figure 2b, revealing that the thermo-oxidative degradation of FUR occurs in six steps, which conforms with the findings of Jamróz et al. [28]. The first thermodegradation step, observed from 25 to 200 °C, is evidently related to the evaporation of water and other low-molecular compounds. In this temperature range, the weight loss of the composites increases with increasing FUR content. The next thermodegradation step, occurring within 200–320 °C, is related to the decomposition of starch moieties, while the height of the second step increases by increasing the TPS concentration in the investigated composites. This degradation step is also influenced by the presence of FUR, while the TGA curve within this temperature region is shifted towards the direction of the FUR decomposition thermogram, i.e., by increasing its content, thermal decomposition begins earlier. Most likely, mass loss in this region is connected to the distribution of the −SO^−^_3_ functionalities containing moieties [28]. Degradation within this temperature region is also influenced by the presence of glycerol plasticizer. As TPS + 10 wt.% FUR contains the highest TPS content, this composite degrades to the greatest extent within 200–320 °C; as a result, at 320 °C, the highest remaining mass is found for the composites with the highest FUR content, namely TPS + 50 wt.% FUR. The next thermal degradation step, within 320–500 °C, is mainly because of FUR degradation. Within this region, it is evident that by increasing the concentration of FUR from 10 to 50 wt.%, the maximum thermo-oxidative degradation peak is shifted towards higher temperatures from ca 360 to 385 °C (Figure 2b), which is probably related to the decomposition of the carbohydrate backbone [28,29]. During the last mass loss step within 500–620 °C, the thermodegradation of oxidation products, developed within the previous mass loss steps, occurs. This temperature region has been associated with the decomposition of aromatic hydrocarbons [28,30]. Interestingly, at the end of the thermogravimetric test, the highest remaining mass was observed for the TPS composite with 50 wt.% FUR, denoting the dominant role of FUR in the formation of a non-combustible, gas-impermeable char layer during the carbonization process. This may denote the possible improvement of the flame retardancy of the composites containing FUR.

### 3.3. Differential Scanning Calorimetry

**Figure 3:**
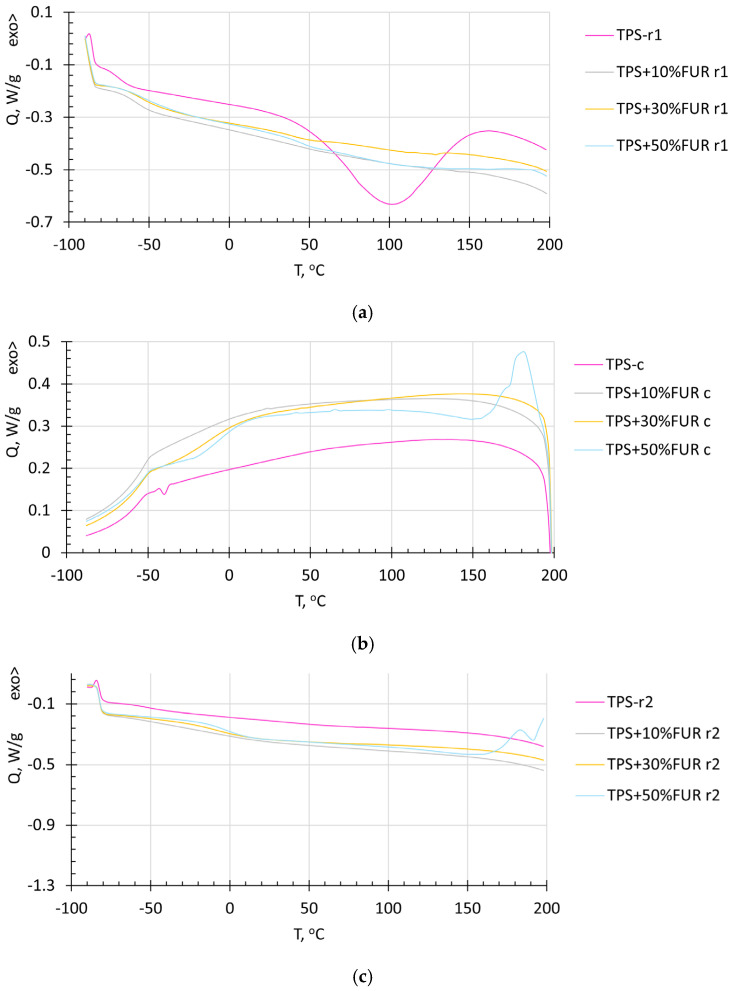
DSC thermograms of TPS and its composites with FUR: (**a**) thermograms from the 1st heating run (marked with the abbreviation r1); (**b**) thermograms from the cooling run (marked with the abbreviation c); (**c**) thermograms from the 2nd heating run (marked with the abbreviation r2).

DSC thermograms of TPS and its composites with FUR collected from the first and second heating runs, as well as the cooling run, are demonstrated in Figure 3. The large endothermic peak, observed in the first-run thermogram of TPS, denotes the presence of a considerable amount of water. A concomitant, broad endothermic effect is also observed for the composite containing the greatest amount of FUR (50 wt.%). This broad endothermic effect is probably related to the presence of FUR and may denote the evaporation of different low-molecular compounds, including water. By decreasing the FUR content, this endothermic effect is considerably reduced, remaining hardly visible for the composite with 10 wt.% FUR. However, in all the thermograms, the low-temperature transition temperature T_LT_, as also reported by other authors [31], is clearly visible, shifting to the direction of the higher temperatures with increasing FUR content: −72 °C for TPS, −56 °C for TPS + 10 wt.% FUR and −52 °C for the TPS composites with 30 and 50 wt.% FUR. This denotes that the interaction between TPS and FUR (most likely due to H bonding) is affected by increasing FUR concentration, while it is well known that a shift in the relaxation transitions of the individual components of polymer blends demonstrates their compatibility.

From the cooling thermograms, a large endothermic effect can be observed for the composite with the highest FUR weight content, denoting that partial degradation of the material occurs. At lower FUR contents, no endothermic effects are observed, demonstrating the larger thermal stability of these composites. From the cooling runs, two thermal transition regions may be observed, particularly for the TPS + 50 wt.% FUR composite. Two relaxation temperatures in 50-to-50 wt.% plasticized starch and carrageenan films have previously been observed by Chang-Bravo, Lopez-Cordoba and Martino, who detected two relaxation transitions at ca −69 °C and −3 °C, relating them to the plasticizer-rich phase and starch-rich phase [32]. This confirms our previous statement about the composites with 50 wt.% FUR demonstrating the lowest compatibility between TPS and FUR, in comparison to the systems with 30 wt.% and, especially, 10 wt.% FUR, which demonstrated less pronounced relaxations over the temperature range −50 to 20 °C.

The second heating run thermograms, in general, confirm the effects observed above. T_LT_ is increased and becomes more visible by increasing FUR content. Similarly, as in the first heating run tests, T_LT_, as observed in the second heating run experiments, increases with increasing FUR content, i.e., −25°C for TPS + 10 wt.% FUR, −20 °C for TPS + 30 wt.% FUR and −10 °C for TPS + 50 wt.% FUR, confirming once more that the highest compatibility between TPS and FUR is found in the composite containing 10 wt.% of FUR. The fact that the second relaxation temperature is not visible in the second-run thermograms is probably related to the temperature change rate being too low (10 °C/min), while it has been previously determined that, if the temperature change rate is lower than 50 °C/min, it may disturb observations of relaxation temperatures in biopolymer systems [33].

### 3.4. Rheological Properties

**Figure 4:**
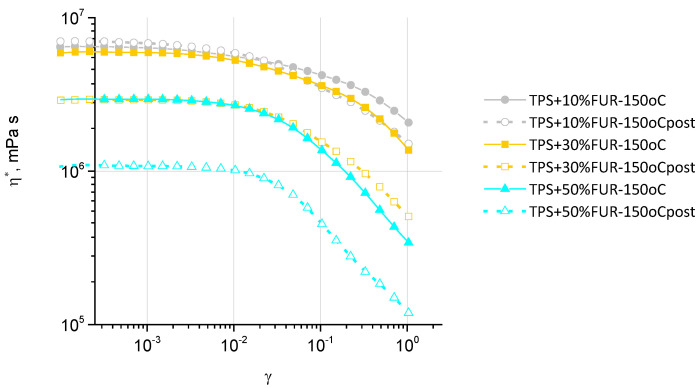
Complex viscosity–strain amplitude sweep relationships for TPS/FUR composites before (closed symbols) and after (open symbols) frequency scan tests.

The complex viscosity η*–strain amplitude sweep relationships of TPS/FUR composites before and after the frequency scan test are shown in Figure 4, revealing that for all of the composites, the linear viscoelasticity region (LVER) is observed below 1% of shear strain, and was therefore selected for further frequency scan tests.

As demonstrated in Figure 4, increasing FUR concentration in the investigated composites causes the complex viscosity η* to decrease; however, if the content of FUR is raised from 10 to 30 wt.%, the decrease in η* is insignificant, contrasting the considerable η* reduction if FUR content is increased from 30 to 50 wt.%. Concomitantly, the η* decrease seen in TPS/FUR composites increases with increasing shear strain, especially when changing FUR content from 30 to 50 wt.%, which denotes increased shear sensitivity. After frequency scan tests, the η* of the composites dropped due to material ageing during the test. However, the η* drop greatly differs: if for TPS + 10 wt.% FUR there is insignificant η* change, showing some deviation generally beyond the LVER, at higher FUR contents, significant differences are observed within the whole shear strain range. This denotes the relatively low η* stability of TPS + 30 wt.% FUR and especially TPS + 50 wt.% FUR at high shear strains, making these composites less suitable for thermoplastic processing.

**Figure 5:**
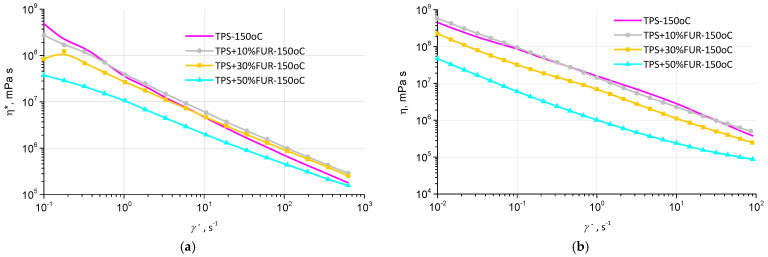
Complex viscosity η* (**a**) and dynamic viscosity η (**b**) of various TPS/FUR composites as functions of shear rate.

The flow curves of TPS/FUR composites in oscillation and rotation modes are demonstrated in Figure 5. As expected, viscosity, measured in both oscillation and rotation modes, decreases with increasing shear rate/frequency, whereas the highest viscosity values are observed for neat TPS as well as its composite with 10 wt.% of FUR, followed by the composites with 30 wt.% of FUR and 50 wt.% of FUR. The higher viscosity values of TPS + 10 wt.% FUR can be explained by the better intermolecular interaction between TPS and FUR, whereas at higher FUR concentrations, due to increased intramolecular interactions between FUR chains, binding between TPS and FUR is partly prevented.

### 3.5. Tensile Properties

**Figure 6:**
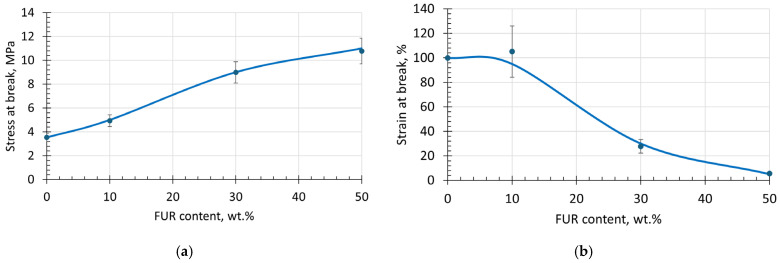
Stress at break (**a**) and strain at break (**b**) of TPS/FUR composites as a function of FUR weight content.

The effect of FUR on the ultimate stress–strain characteristics of TPS/FUR composites is depicted in Figure 6 and reveals that by increasing FUR content until 50 wt.%, the average values of the stress at break σ_b_ increase almost three times from 3.5 MPa to 10.8 MPa; however, this is accompanied by a considerable decrease in strain at break ε_b_. Interestingly, the TPS composite with 10 wt.% FUR maintains almost the same ε_b_ as the matrix; meanwhile, the σ_b_ of this composite is ca 20% larger than that for neat TPS. Increasing FUR content above 10 wt.% leads to a considerable decrease in ε_b_ until reaching ca 25–30%. At this level, however, the σ_b_ of the composite reaches almost 10 MPa, which is more than two times higher than for the neat TPS matrix. By increasing the FUR concentration even more, the rates of σ_b_ rise and ε_b_ decrease diminish. The observed changes are most probably related to the interplay between the formation of intramolecular H bonds between FUR and intermolecular H bonds between plasticized starch and FUR at elevated temperatures (ca 80 °C) during the first manufacturing stage of the investigated composites. At higher FUR content, the role of intramolecular bonding within FUR becomes more important, leading to increased strength on the one hand and decreased ultimate elongation on the other hand, simultaneously denoting the least compatibility within the TPS + 50 wt.% FUR composite. As may be concluded from Figure 6, with respect to mechanical properties, 10–30 wt.% of FUR in TPS could be suggested as the most preferable choice.

## 4. Conclusions

The results of the current research on waste red seaweed-extracted furcellaran *F. lumbricalis* (FUR) demonstrate the possibilities for obtaining thermoplastic starch (TPS)-based biodegradable and biobased composites, revealing several interesting aspects.

For the first time, it was possible to obtain thermoplastically processable TPS-based composites containing FUR concentrations of up to 50 wt.% by using an ultrasound-assisted two-step manufacturing approach.

As demonstrated by the FTIR spectra, intramolecular interaction in FUR becomes stronger by increasing its content in the investigated composites, simultaneously leading to decreased compatibility with TPS. Because of both intra- and intermolecular interactions (most likely due to the formation of H-bonds), the highest stress at break is observed for the TPS composite with 50 wt.% FUR (up to 12 MPa, which is almost three times higher than for the neat TPS matrix); however, the considerably decreased strain at break (ca 10%) must be accounted for. Meanwhile, the TPS composite with 10 wt.% of FUR maintained almost the same ultimate deformation as TPS (100%), but showed 1.4 times larger strength at break (5 MPa).

In respects to rheological properties, the addition of 10 wt.% of FUR had practically no influence on the melt viscosity of TPS/FUR composites, whereas increasing FUR content up to 50 wt.% caused a decrease in viscosity because of the lower thermal stability of FUR, as well as the decreased compatibility between TPS and FUR.

In conclusion, the TPS + 10 wt.% FUR composite demonstrated the highest thermal resistance as well as the most stable melt-processing behavior, allowing us to suggest that this composite is the most suitable for thermoplastic processing.

## Data Availability

The raw data supporting the conclusions of this article will be made available by the authors on request.
